# Structural analysis of customized 3D printed plate for pelvic bone by comparison with conventional plate based on bending process

**DOI:** 10.1038/s41598-023-37433-1

**Published:** 2023-06-29

**Authors:** Woo-Lam Jo, Yang-Guk Chung, Seung-Han Shin, Jae-hak Lim, Moo-Sub Kim, Do-Kun Yoon

**Affiliations:** 1 Department of Orthopaedic Surgery, Seoul St. Mary’s Hospital, College of Medicine, The Catholic University of Korea, Seoul, 222, Banpo-Daero, Seocho-Gu, Korea; 2 Industrial R&D Center, KAVILAB Co. Ltd., 06693 Seoul, Republic of Korea

**Keywords:** Computational biology and bioinformatics, Anatomy

## Abstract

Pelvic bone fracture is highly complex, and its anatomical reduction is difficult. Therefore, patient-specific customized plates have been developed using three-dimensional (3D) printing technology and are being increasingly used. In this study, the reduction status in five representative pelvic fracture models was compared between two groups: the 3D printing plate (3DP) group using a patient-specific 3D printed plate after virtual reduction and the conventional plate (CP) group using a conventional plate by manual bending. The 3DP and CP groups included 10 and 5 cases, respectively. The fractured models were reduced virtually and their non-locking metal plates were customized using 3D printing. The process of contouring the conventional plates to fit the contact surface of the bone with the bending tool was conducted by an experienced pelvic bone trauma surgeon. The reduction and fixation achieved using the two different plate groups was compared, and the significance of differences in the results was analyzed using paired t-tests, after verifying the normality of data distribution. The vertex distances between the surface of the bone and the contact surface of the plate were significantly lower in the 3DP group than in the CP group (0.407 ± 0.342 and 2.195 ± 1.643, respectively, P = 0.008). Length and angular variations, which are measurements of the reduction state, were also lower in the 3DP group than in the CP group (length variation: 3.211 ± 2.497 and 5.493 ± 3.609, respectively, P = 0.051; angular variation: 2.958 ± 1.977 and 4.352 ± 1.947, respectively, P = 0.037). The customized 3D printed plate in the virtual reduction model provided a highly accurate reduction of pelvic bone fractures, suggesting that the customized 3D printed plate may help ensure easy and accurate reduction.

## Introduction

Pelvic and acetabular fractures are relatively rare, accounting for approximately 1.5% of all fractures. However, they are major injuries and can be life-threatening in severe cases^1–3^, with an overall mortality of 6–35%^4–6^. In Sweden, the incidence of pelvic and acetabular fractures increased markedly from 2001 to 2016^7^. Additionally, in France, the incidence of fracture has rapidly increased in the young as well as older individuals, who may require surgery^8^. The incidence has also seemingly increased in other developed countries, while the mortality has declined^9^.

In addition to the pelvic ring injuries, acetabular fractures affect the hip joints, necessitating functional recovery. Owing to the development of surgical approaches and basic supporting techniques, surgical treatments are frequently performed for rapid functional recovery.

Anatomical reduction is important for early fusion and quick functional recovery. However, unlike other long bones, the pelvis has a complex shape, causing difficulty inperforming anatomical reduction. An essential yet challenging requirement for anatomical reduction is that the plate must be bent precisely to fit the contour of the original pelvis. Recently, anatomical plates based on numerous patient data have become commercially available; however, they do not guarantee anatomical reduction in all patients^10,11^.

Patient-specific, customized plates created using three-dimensional (3D) printing technology have been developed and are increasingly being used^12–15^. Case-specific studies on the tibial sawbone shaft have reported favorable results in terms of the restoration of near-normal anatomy in fractures^16^. However, the applicability of this method on bones with complex anatomy, such as the pelvic bone, has not yet been reported.

In this study, we compared the reduction status in the pelvic fracture model between two groups: one using patient-specific 3D printed plates after virtual reduction (VR) and the other using conventional plates created by manual bending. We hypothesized that patient-specific 3D printing plates can provide better fracture reduction than conventional plates.

## Materials and methods

### Preparation of materials

For the experimental verification of the precision of the 3D printed pelvic plate, 15 (seven left-sided and eight right-sided) intact pelvic ORTHObones (ORTHObones Premium Right/Left Half Pelvis, 3B Scientific, Germany, Hamburg) were prepared. All cases were analyzed using computed tomography (CT) (120 kVp at 100 mAs; Siemens, SOMATOM Definitions AS+, Germany, Munich). However, the two steps before CT scanning were preferentially progressed for the experiment.

**Figure 1:**
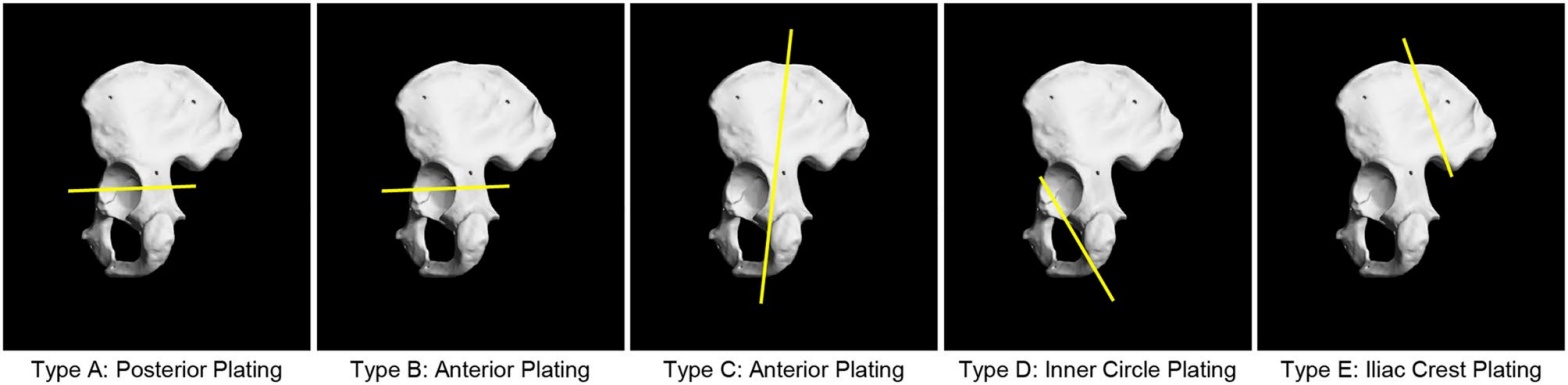
Fracture line set-up and templating plan according to types of fractures (five types).

**Figure 2:**
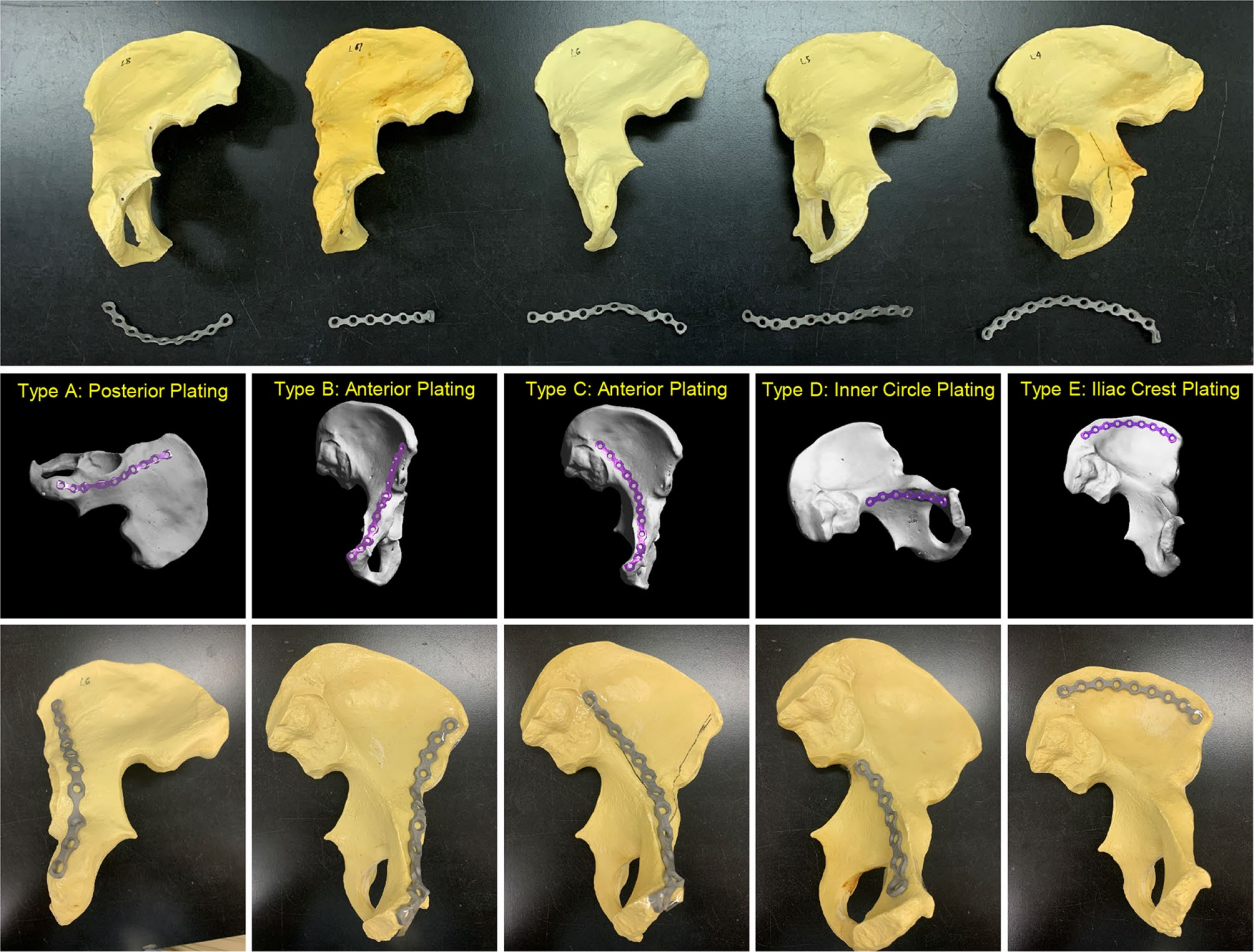
Final designs of the customized plates according to fracture type and fabricated plates. Type A: transverse fracture with posterior plating. Type B: transverse fracture with anterior plating. Type C: anterior column fracture with anterior plating. Type D: posterior column fracture with anterior plating. Type E: iliac wing fracture with iliac crest plating.

First, we drew a single line on each intact bone to indicate the fracture lines of the five representative fracture types. The fracture was presented as a single fracture that generated two fracture fragments. Second, we considered five types of fractures to representat the patterns at the pelvis (Figs. 1 and 2) and drilled six cortical marking holes (three on each fragment) on each ORTHObone according to the line drawn, todetermine the surface. The three cortical marking holes on each bone fragment allowed us to set a composition of a virtual plane of two vectors, and CT images were acquired with 491.52 mm × 491.52 mm × 1,157.25 mm field of view size, according to the digital imaging and communications in medicine (DICOM) format(pixel size: 0.96 mm × 0.96 mm, pixel matrix: 512 × 512, slice thickness: 0.75 mm, number of slices: 1,543), according to the digital imaging and communications in medicine (DICOM) format. After the first CT scan, a ssurgical electronic saw was used to make cracks according to the line drawn, and the ORTHObones were ultimately fractured using physical force based on the splitting. The second CT scanning was performed with the same condition as the first scanning. All DICOM images segmented for a part of the bone were reconstructed as 3D images using the dedicated medical image processing software, MIMICS (Ver 21.0, Materialise, Belgium, Leuven).

### Customized design and fabrication of the plate

**Figure 3:**
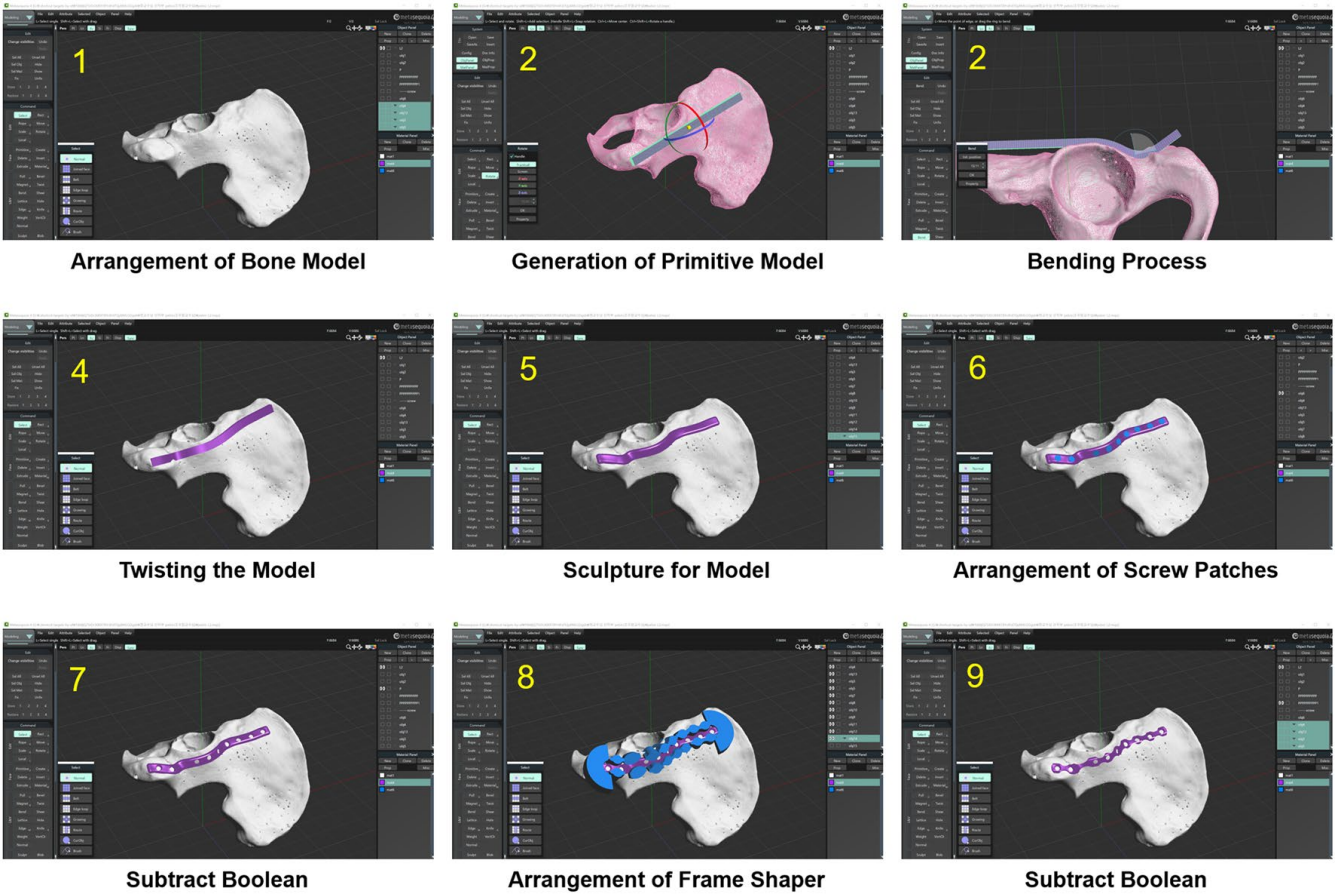
Modelling tool screen during the custom plate design process; (1) Arrangement of Bone Model: Arrangement of the 3D bone model in the 3D modeling tool to design work, (2) Generation of Primitive Model: Generation of the primitive structure which will be a plate frame in the 3D modeling tool, (3) Bending Process: The graphically bending for primitive structure to mold the external shape in the 3D modeling tool, (4) Twisting the Model: The graphically twisting for primitive structure to mold the external shape in the 3D modeling tool, (5) Sculpture for Model: Smoothing process for the surface of the plate frame, (6) Arrangement of Screw Patches: The process of the making hole for screw using the Boolean function and the dedicated patches, (7) Subtract Boolean: The subtract Boolean between the screw patches and plate frame, and between the plate frame and 3D bone model, (8) Arrangement of Frame Shaper: the location of frame shaper which can mold the specific external shape of the plate, (9) Subtract Boolean: The subtract Boolean between the frame shaper and plate frame.

The first step in designing the patient-specific 3D printed plates was a virtual reduction of the fractured bone. The 3D reconstructed bone images were accessed using the 3D modeling tool Metasequoia 4 (Tetraface, Ver 4.7.0, Japan, Tokyo), and the virtual reduction of the fractured bone was conducted by relocating the 3D object for the bone fragment to its proper position according to the fracture line. Thereafter, the designing of plate and arrangement of its initial position on the 3D object of the reduced bone were completed, and the customized design of the plate was conducted using several functions of Metasequoia 4, including bending, twisting, and the Boolean function. The contact surface (with the ORTHObone) of the plate was designed using Boolean functions to facilitate accurate contact between the bone surface and plate. The designed plates were prepared using a powder bed fusion-type 3D printer (DMP 350, 3D Systems, USA, SC) and titanium powder (Ti-6AI-4V alloy_Grade23). Post processing, the supporter was removed, surface finishing was completed using a handpiece, and blasting processes using ceramic microbeads were performed (Fig. 3).

The average time for use of the 3D modeling tool including final modification was 3 h for each plate. Although the first design of the customized plates (draft) takes relatively short time as 1 h per each plate, and many times of modifications made them better. The total time for the output of the plates (correctly, 10 plates) on the metal 3D printer was 8 h with one printing operation. The fabrication for all plates including post-processing and delivery took 2 days, and the total cost of the fabrication charged about 5000 USD.

In this study, 10 customized plates (two cases each for five types) were designed and fabricated. Conventional plates (CPs) were used for comparison using the remaining five cases according to the type of fracture. The use of CP with bending process for contouring depends on the skill and experience of the surgeon. Hence, the performance of the CP in this study cannot be absolute indicators, and the performance of CP in this study can be close to the average level of contouring results by the trained orthopeadic surgeon. For this reason, we only used the best CP models according to type with manual contouring process.

The ORTHObones were tagged as ‘Right’ and ‘Left’, wherein R1 to R7 denoted the right cases, and from L1 to L8 denoted the left cases. All fracture types involved both cases for the 3D printed plates (3DP) and one case for the conventional plates (CP). Type A (horizontal fracture with posterior plating) included L2 for CP, and L6 and R5 for 3DP, while Type B (horizontal fracture with anterior plating) included L5 for CP, and R1 and R4 for 3DP. Moreover, Type C (vertical fracture with anterior plating) included L1 for CP, and L4 and R3 for 3DP, while Type D (horizontal fracture with inner circle plating) included L7 for CP, and L3 and R6 for 3DP. Lastly, Type E (vertical fracture with iliac crest plating) included R7 for CP, and L8 and R2 for 3DP. The Matta pelvic plates with flex type (PRO Curved and Straight Plate from 7 to 10*, Stryker, USA, MI) were used for ifixation in the CP cases.

### Reduction, fixation, and 3D scanning

**Figure 4:**
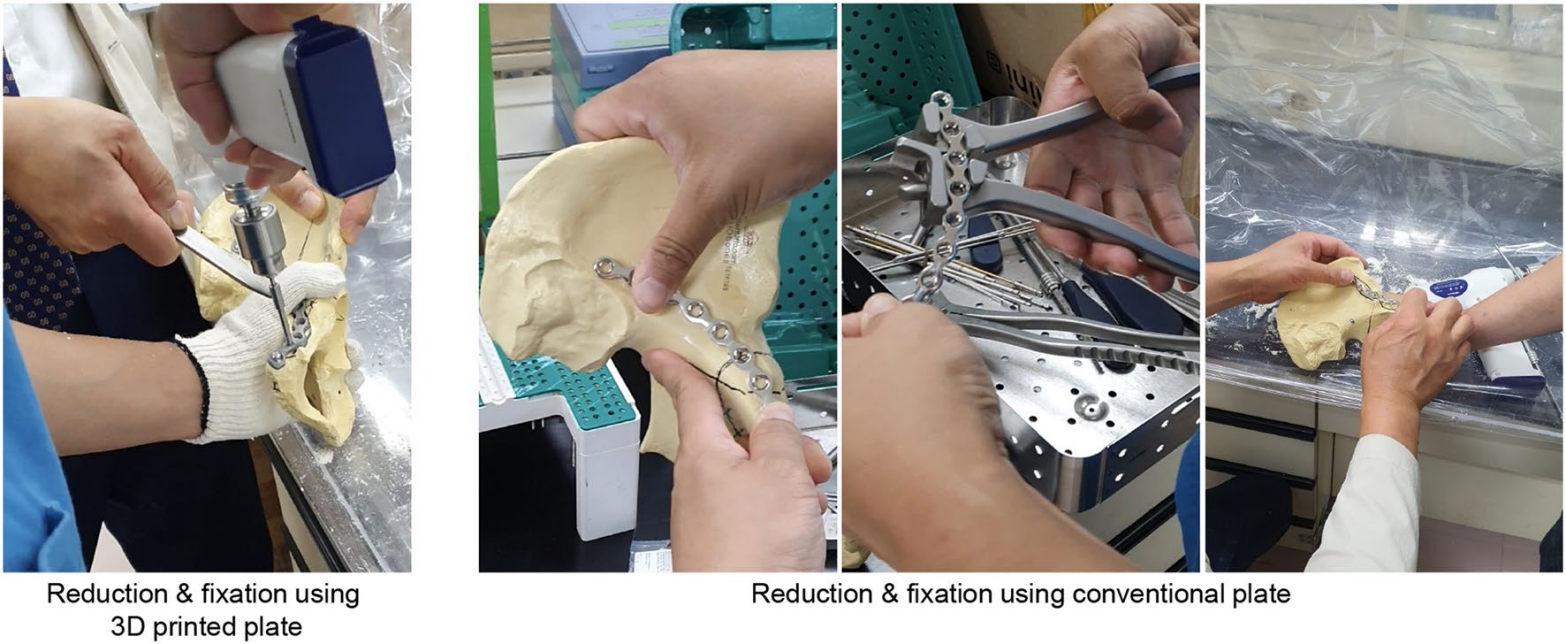
Reduction and fixation using conventional (CP; right side) and three-dimensional printed plates (3DP; left side). The CP group included the contouring of plate using the bending tool after temporary reduction.

After preparing all the plates, reduction and fixation were performed by an experienced orthopedic surgeon with more than 10 years of practice. In the CP cases, the plates were contoured by an experienced pelvic bone trauma surgeon to fit the contact surface of the bone using a bending tool (Fig. 4). This process was repeated 5–6 times to obtain satisfactory optimal shapes after temporary reduction. It usually takes 3 to 5 min to bend the plate according to the shape of the pelvic bone at first. And it takes about 5 min for 5–6 corrections, so the average time is within 10 min per plate. Subsequently, the contoured plate was placed on the bone surface and fixed with cortical screws.

In the 3DP cases, reduction and fixation were conducted without the contouring process due to the high degree of conformity between the surface of the bone and the contact surface of the plate. The fixed ORTHObones were then scanned using the 3D scanner with resolution of 29 microns (0.029 mm), using a 70 mm measurement volume (ATOS ScanPort, GOM, Germany, Braunschweig). However, accurate measurement using the CT image is not practically possible because the metal plate causes a metal artifact in the CT image. The scanned images were exported in stereolithography (STL) format, which was sbseuently accessed using the Metasequoia 4 system.

### Image registration and 3D measurement

To compare the performance of the reduction and fixation between the two kinds of plates (CP and 3DP), the original 3D bone models before the fracture were registered to the 3D scanned images using the method of the iterative closest point (ICP), which is one of the functions in Metasequoia 4. The criteria for the ICP included the coordination of the three center points of the drilled cortical marking holes on each bone fragment. The larger of the two fragments was designated as the principal fragment. When the other fragment underwent displacement during reduction and fixation, the positions of the three points on this other fragment did not correspond with the positions of the three points on the original bone.

**Figure 5:**
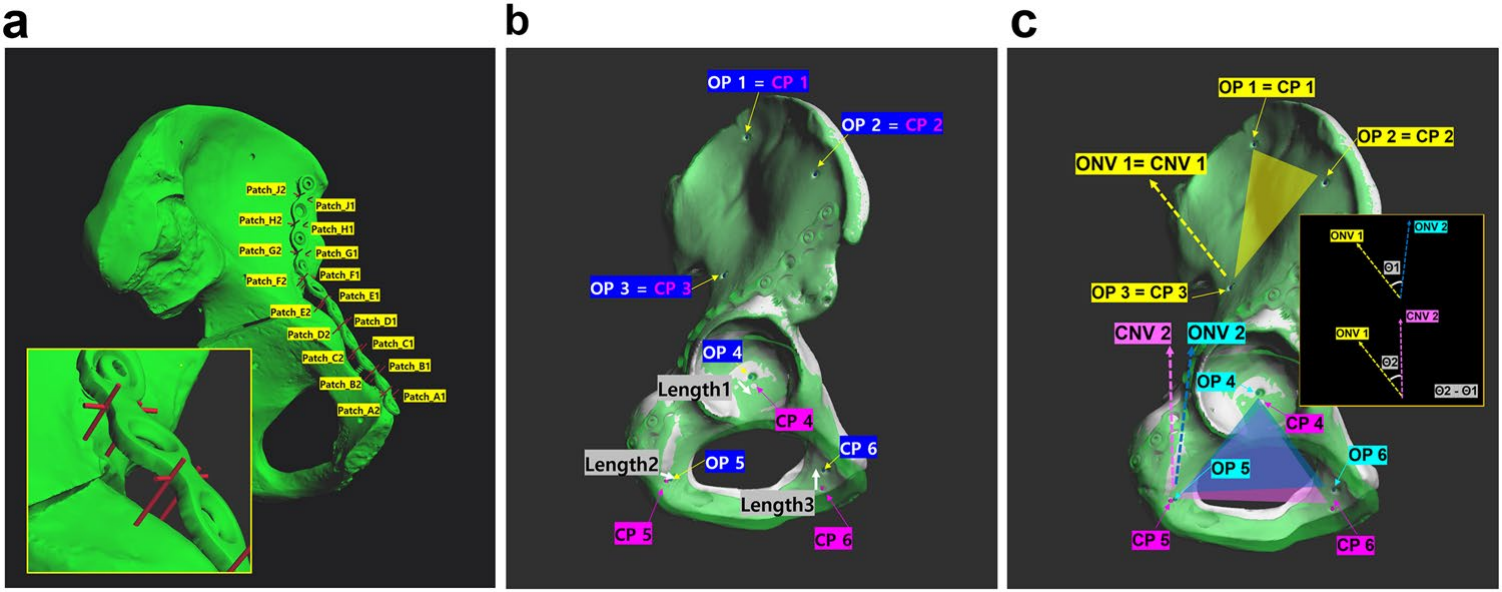
Concept figure for three-dimensional (3D) measurement. (**a**) The vertex distances were measured using ‘T’ type patches, which were tagged in alphabetical and numerical order (1: right side, 2: left side). (**b**) Three kinds of lengths were measured. The original points (OP) from 1 to 3 were matched with the changed points (CP) from 1 to 3. The lengths 1, 2, and 3 indicate the distances between OP 4 and CP 4, OP 5 and CP 5, OP 6 and CP 6, respectively. (**c**) The angulation variation is measured by calculating the difference in angles between Θ1 and Θ2. Θ1 is the 3D angle formed between original normal vectors (ONV) 1 and ONV 2, and Θ2 is the 3D angle formed between ONV 1 and changed normal vector (CNV) 2. Each normal vector originated from a plane formed by three points.

Reproducibility by plating in this study were compared using four measurement methods. First, the vertex distances between the surface of the bone and the contact surface of the plate were measured on the Metasequoia 4 system. To easily locate the vertices, we applied the ‘T’ type patch, which is perpendicularly arranged to the side surface of the plate. The patch’s head laterally penetrated the center of the side of the plate (thickness), and the vertex distance was measured by subtracting half of the plate thickness from the original length of the patch’s leg. Figure 5a shows the arrangement of ‘T’ type patches for the measurement of the vertex distance. Although these patches are not visible in the 3D modeling tool, the shapes of the patches were included in the figure for clearer understanding.

The second method of measurement used length variation, which describes the moving distance between the original point and the point changed during reduction and fixation. The three points on the larger fragment were matched with the original points using the original image on the system; therefore, three types of lengths were obtained by the movement of the three points. The position of the 3D scanned image in the 3D modeling tool was transformed to absolutely register to the original 3D bone model, the three pairs of coordinates (original points on the original bone, and changed points on the 3D scanned bone) were extracted and the distance between the pair of coordinates was calculated (Fig. 5b).

Third, the 3D angulation variation was measured using cortical marking holes, similar to that in the second method. The three points can form a plane and its normal vector, which is the vector perpendicular with the plane; therefore, there were three types of normal vectors: original normal vector 1 (ONV 1, matched with changed normal vector 1 [CNV 1]), ONV 2, and CNV 2. The two types of formed angles (ONV 1 with ONV 2, and ONV 1 with CNV 2) were then calculated. Figure 5c shows the setup of the planes and normal vectors. We calculated the difference between these two angles through vector calculation, which cannot be simply subtracted owing to the presence of the 3D angle.

### Statistical analysis

All statistical analyses were conducted using the Statistics and Machine Learning Toolbox on MATLAB (R2021b, MathWorks, USA). The results have been demonstrated as means and standard deviations, and the significance in the differences of the results was analyzed using paired t-tests, after verifying the normality of data distribution. P < 0.05 was considered statistically significant.

## Results

### Vertex distance

The vertex distances between the surface of the bone and the contact surface of the plate were measured and found to be longer for the conventional plate than for the 3D printed plate in all fracture models. The vertex distance indicates the degree of floatation between the plate and the pelvic bone surface, and its value increases with the decrease in anatomical reduction or conforming congruence. The difference was found to be particularly large in the posterior plating group with a transverse fracture. The average value of the vertex distance was significantly shorter in the 3DP group than in the CP group (0.407 ± 0.342 and 2.195 ± 1.643, respectively, P = 0.008).

### Length variation

**Figure 6:**
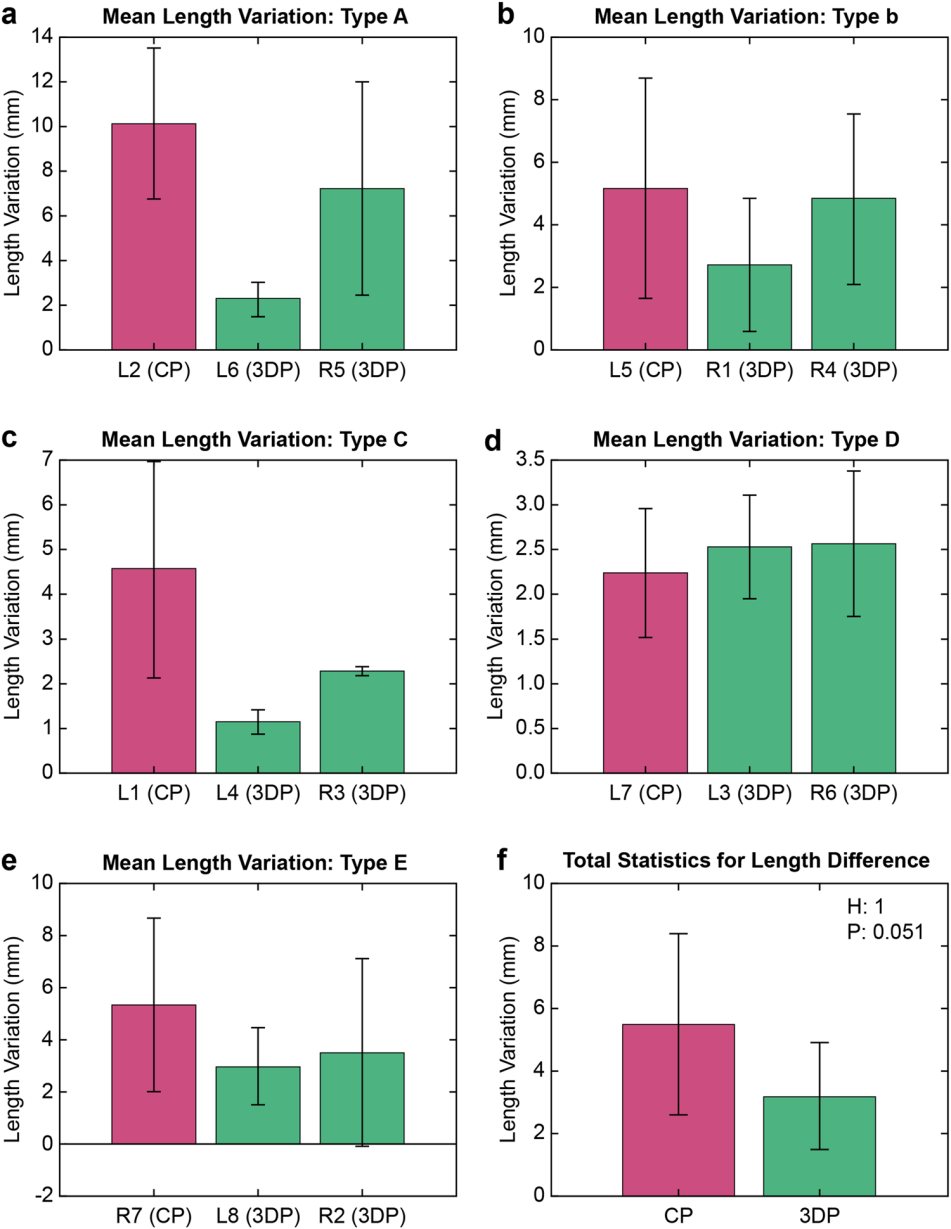
Mean length variation according to fracture type. The types have been classified by the fracture pattern. The Types, from A to E, are matched with (**a**)–(**e**). (**f**) Shows the sum of the length variation between the conventional and three-dimensional plate groups.

Length variation is an indicator that can determine how well the fracture reduction has been achieved. The length variation was generally larger in the CP group than in the 3DP group; however, the difference in total length variation was not statistically significant (5.493 ± 3.609 and 3.211 ± 2.497, respectively, P = 0.051) (Fig. 6).

### Angulation variation

**Figure 7:**
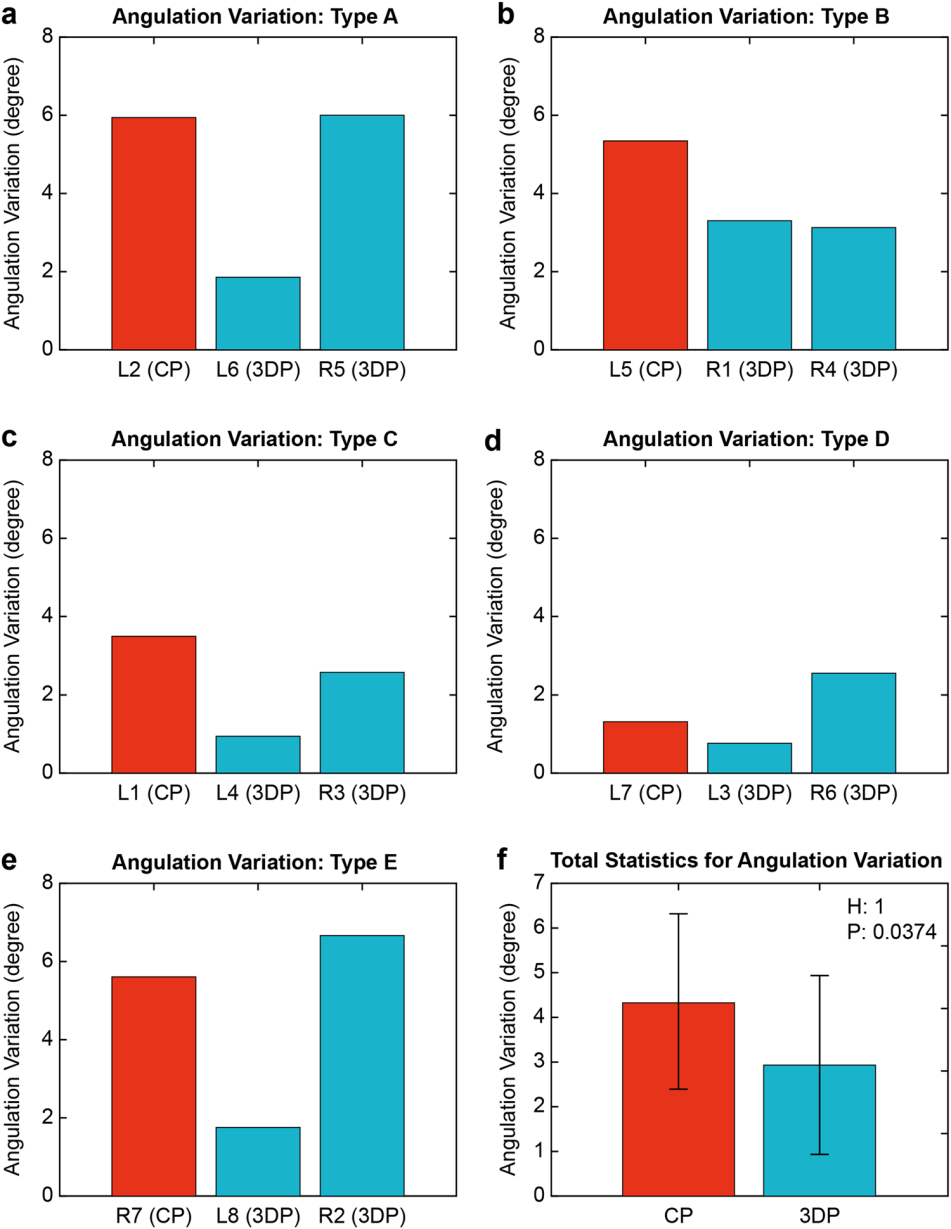
Angulation variation according to fracture type. The types have been classified by the fracture pattern. The Types, from A to E, are matched with (**a**)–(**e**). (**f**) Shows the sum of the angulation variation between the conventional and three-dimensional plate groups.

The angular variation was significantly larger in the CP group than in the 3DP group (4.352 ± 1.947 and 2.958 ± 1.977, respectively, P = 0.0374) (Fig. 7).

## Discussion

The main finding of the current study is that it is possible to reduce fractured bones to approximately their original anatomy with 3D printed custom-made plates based on virtual reduction. In general, the acceptable range of fracture reduction alignment is evaluated by length, angulation, and rotation^17,18^. In addition, there is a method to evaluate the reduction status according to the gap of acetabular fracture^19^. Compared with the conventional plates, customized 3D printing plates facilitated more effective and accurate reduction in complex pelvic bone fractures^20^.

Currently, in pelvic and acetabular fracture surgery, fracture reduction is performed manually, and the metal plate is manually bent and aligned to fit the bone. Unlike long bones of the extremities, the pelvic bone is complexly contoured and is located under the internal organs and close to the neurovascular structures; therefore, it is very difficult to adjust the plate to the desired shape. Thus, the conventional method is time-consuming and leads to excessive bleeding during the process, and the congruent shape to the pelvic bone cannot be obtained even after several bending processes in some cases. Therefore, the results of fracture reduction can vary depending on the operator's skill and technique.

Therefore, anatomical plates were developed based on the population’s pelvic bone data and have been made commercially available. An anatomical suprapectineal QLS plate (Stryker®, Selzach, Switzerland) was recently introduced and has been widely used in complex acetabular fracture treatments. Although the use of these plates has several advantages, such as improved quality of reduction and shorter operation time, it still cannot match some pelvic bone fractures owing to variation in in bone size and shape and fracture location^11^.

Tactile modeling with 3D printing of the fractured bone has been used during presurgical practise and precontouring of the plate; however, the overall procedure still entails a manual reduction of the tactile model, and bending and twisting of the plate by the operator^21,22^.

Another method of forming a plate that has recently emerged involves mirroring the normal bone on the contralateral side. Wang et al. reported the preparation of 3D printed patient-specific pelvic fracture plates based on mirrored 3D models of the contralateral pelvis^23^. However, this method cannot be used in case of a bilateral fracture or with a history of previous trauma on the opposite side. Additionally, the structures of the left and right pelvis can be minimally different.

In this study, we used a virtually reduced model as the basis of a patient-specific 3D plate and achieved approximately 30% more accurate fracture reduction in terms of length and angular variations than those obtained with the existing manual bending plate. As a result, a length variation value of 3 mm or less was obtained on average in 3DP. This is a value reduced by about 30% compared to CP, which is not perfect, but is within the acceptable fracture gap range.

VR is advantageous over manual reduction during surgery, which requires the operator to visualize only the exposed portions of the bone or using fluoroscopy. Furthermore, we can obtain a more accurate reduction status without any concern regarding the difference between the left and right pelvic bones by not using the mirroring of the contralateral pelvic bone. The VR-based 3D plate itself can be a reduction guide during surgery, and the operator can both reduce the fracture and determine the alignment by contacting the bone fragments to the plate. However, the compression between the fracture fragments cannot be deliberately obtained by under- or over-bending the plate. The compression at the fracture site is more important in the pelvic bone than in the long bones of extremities; therefore, a modification of the design of the 3D printed plate to provide compression force to a fracture site is necessary. This is considered to be a larger disadvantage in pelvic bones than in long bones. Therefore, further studies are needed to investigate plates that can provide compression to a fractured site.

And fracture gap increases unpredictably after plate fixation in some case. This can occur in areas other than the surface on which the plate is placed, especially when using CP plates. This is affected by location of the bending point and the degree of bending of the plate. This is because it is difficult to accurately make the contour of the pelvic bone hand-made.

Additionally, studies with larger sample sizes and more complex fracture models are required.

Our study had several limitations. First, the sample size was very small, with three cases per group. Second, fracture models were handmade using an oscillating saw, which can result in bone loss due to the thickness of the saw and affect the reduction status. Since the oscillating saw has a straight cut, the reduction of fractures can be more accurate in experiments, but less accurate in real with more complex fracture patterns. Especially, in cases with compacted bone, missing or necrotic smaller fragments. Third, the experiment was performed on only two fragment fractures; more complex fractures, which have more difficulty with regard to reduction, were not included. Finally, we did not consider muscle contracture or soft tissue tension in this study.

## Conclusions

In conclusion, we obtained more precise reduction in pelvic bone fractures using customized 3D printed plate in the virtual reduction model. Thus, the customized 3D printed plate may be used, with the benefits of easy, accurate reduction.

## Data Availability

The datasets generated during and/or analysed during the current study are available from the corresponding author on reasonable request.

## Abbreviations

3D: Three-dimensional
3DP: Three-dimensional printing plate
CNV: Changed normal vector
CP: Conventional plate
CT: Computed tomography
DICOM: Digital imaging and communications in medicine
ICP: Iterative closest point
ONV: Original normal vector
STL: Stereolithography
