# Author Correction: Structural analysis of customized 3D printed plate for pelvic bone by comparison with conventional plate based on bending process

**DOI:** 10.1038/s41598-023-49800-z

**Published:** 2023-12-15

**Authors:** Woo-Lam Jo, Yang-Guk Chung, Seung-Han Shin, Jae-hak Lim, Moo-Sub Kim, Do-Kun Yoon

**Affiliations:** 1 Department of Orthopaedic Surgery, Seoul St. Mary’s Hospital, College of Medicine, The Catholic University of Korea, 222, Banpo-Daero, Seocho-Gu, Seoul, Republic of Korea; 2 Industrial R&D Center, KAVILAB Co. Ltd., 06693 Seoul, Republic of Korea

Correction to: *Scientific Reports* 10.1038/s41598-023-37433-1, published online 29 June 2023

The original version of this Article contained an error in Affiliation 1, which was incorrectly given as ‘Department of Orthopaedic Surgery, The Catholic University of Seoul St. Mary’s Hospital, 222, Banpo-Daero, Seocho-Gu, Seoul, Republic of Korea’. The correct affiliation is listed below:

Department of Orthopaedic Surgery, Seoul St. Mary’s Hospital, College of Medicine, The Catholic University of Korea, Seoul, 222, Banpo-Daero, Seocho-Gu, Korea

The original Article has been corrected.

